# From ‘Shark-Week’ to ‘Mangina’: An Analysis of Words Used by People of Marginalized Sexual Orientations and/or Gender Identities to Replace Common Sexual and Reproductive Health Terms

**DOI:** 10.1089/heq.2021.0022

**Published:** 2021-09-30

**Authors:** Sachiko Ragosta, Juno Obedin-Maliver, Laura Fix, Ari Stoeffler, Jen Hastings, Matthew R. Capriotti, Annesa Flentje, Micah E. Lubensky, Mitchell R. Lunn, Heidi Moseson

**Affiliations:** ^1^ Ibis Reproductive Health, Oakland, California, USA.; ^2^ Department of Obstetrics and Gynecology, Stanford University School of Medicine, Stanford, California, USA.; ^3^ The PRIDE Study/PRIDEnet, Stanford University School of Medicine, Stanford, California, USA.; ^4^ Ibis Reproductive Health, Cambridge, Massachusetts, USA.; ^5^ Planned Parenthood League of Massachusetts, Boston, Massachusetts, USA.; ^6^ Department of Family and Community Medicine, University of California San Francisco, San Francisco, California, USA.; ^7^ Department of Psychology, San José State University, San Jose, California, USA.; ^8^ Department of Community Health Systems, University of California, San Francisco, San Francisco, California, USA.; ^9^ Alliance Health Project, Department of Psychiatry, University of California, San Francisco, San Francisco, California, USA.; ^10^ Division of Nephrology, Department of Medicine, Stanford University School of Medicine, Stanford, California, USA.

**Keywords:** gender inclusive, sexual and gender minorities, transgender persons, gender-affirming, patient-centered care

## Abstract

**Purpose:** To explore sexual and reproductive health (SRH)-related word-use among sexual and gender minority (SGM) individuals in the United States.

**Methods:** In 2019, we fielded an online quantitative survey on the SRH experiences of SGM adults. Eligible participants included transgender, nonbinary, and gender-expansive (TGE) people assigned female or intersex at birth, and cisgender sexual minority women (CSMW) in the United States. The survey asked participants to indicate if they used each of nine SRH terms, and if not, to provide the word(s) they used. We analyzed patterns in replacement words provided by respondents and tested for differences by gender category with tests of proportions.

**Results:** Among 1704 TGE and 1370 CSMW respondents, 613 (36%) TGE respondents and 92 (7%) CSMW respondents replaced at least 1 SRH term (*p*-for-difference <0.001). Many (23%) replacement words/phrases were entirely unique. For six out of the nine terms, TGE respondents indicated that use of the provided term would depend on the context, the term did not apply to them, or they did not have a replacement word/phrase that worked for them.

**Conclusions:** SRH terms commonly used in clinical and research settings cause discomfort and dysphoria among some SGM individuals. To address inequities in access to and quality of SRH care among SGM individuals, and to overcome long standing fear of mistreatment in clinical settings, more intentional word-use and elicitation from providers and researchers could increase the quality and affirming nature of clinical and research experiences for SGM people.

## Introduction

Language powerfully influences the ways individuals perceive and experience the world.^1^ Word choice can erase or simplify certain experiences, or conversely, validate and amplify experiences.^2,3^ Unfortunately, in the sexual and reproductive health (SRH) field, there is a long history of cisnormative and heteronormative language in both clinical and research spaces that can alienate sexual and gender minority (SGM) individuals.^4^ “Cisnormative” describes the assumption that all people are cisgender, that is, they identify with the gender assumed of their sex assigned at birth. “Heteronormative” refers to the assumption that all people are heterosexual or straight. SGM is an umbrella term for individuals whose experience falls outside of cisgender and heterosexual experiences. An example of these assumptions in clinical and research spaces is the National Survey of Family Growth (NSFG) from 2015 to 2017, which divided participants into “female” and “male,” and referred to “female” participants' partners exclusively with he/him pronouns.^5^

Beyond alienating SGM people, these assumptions undermine the rigor of research and the quality of clinical care. In research spaces, these assumptions can induce selection and measurement biases through inadequate definition of study eligibility criteria and insufficient specificity in definition of certain health behaviors or experiences.^3,6–8^ In clinical care, these assumptions inform the accuracy of language used by providers or health facilities and may contribute to gender dysphoria (distress related to one's gender identity as it relates to their sex assigned at birth and/or how their gender is perceived), reduce people's comfort in seeking care and/or disclosing health behaviors, and impact the appropriateness and quality of care provided.^3,6,9,10^

Prior research indicates that transgender people evaluate health care providers on use of language and will seek out or avoid providers according to the language that affirms their experiences.^7^ In response to cisnormative and heteronormative language, transgender and nonbinary individuals have adopted language to affirm their identities.^2^ Yet, one formative study found that most of the 1788 transgender and nonbinary respondents had not been asked about language preferences for their bodies by their providers, despite wanting to be asked.^11^ One approach to avoiding these issues is for researchers and clinicians to elicit person-centered language from individuals, potentially allowing for greater inclusivity and precision in research and clinical care.^12^ To explore this understudied topic, we set out to measure SRH-related word-use among SGM individuals. Specifically, we aimed to ascertain how many study participants use standard SRH-related medical terms versus their own words, and what those words are through a novel, online questionnaire. Better understanding of word-use among SGM individuals may contribute to improved health equity by creating more affirming research and clinical environments where SGM individuals may feel more comfortable seeking care and disclosing relevant health information, and where providers and researchers will better understand the unique needs of these populations.^3,11^

## Methods

### Study population

Eligible participants included SGM individuals assigned female or intersex at birth, transgender, nonbinary, and gender-expansive (TGE) individuals or cisgender sexual minority women (CSMW), who resided in the United States or its territories, were 18 years or older, and were able to read and understand English. Participants were recruited through social media, email lists, and announcements at community events, and through The Population Research in Identity and Disparities for Equality (PRIDE) Study,^13^ an online national prospective cohort study of SGM adults. Participants in The PRIDE Study had consented to be part of the longitudinal cohort, whereas participants recruited outside of The PRIDE Study agreed to participate in this single survey. Recruitment materials invited interested individuals to participate in an online quantitative survey covering a broad range of SRH topics.^12^

### Measures

Participants completed an online survey, fielded between May and September 2019 through Qualtrics (Qualtrics LLC; Provo, UT), which included several previously described SRH domains.^12^ To measure the primary outcome, we designed the survey to allow use of respondent-provided words and phrases in place of standard SRH terms. To calibrate term meaning, we provided gender-neutral definitions for nine medical terms: “abortion,” “birth control,” “breasts,” “penis,” “period,” “pregnant,” “sperm,” “uterus,” and “vagina.” After reading each definition, the survey prompted all respondents to indicate whether they used that word, did not use that word, or preferred not to say. Respondents who indicated use of another word had to provide their replacement word/phrase to continue. After data were collected separately for each term, these replacement words were substituted for the medical terms throughout the remaining questions and answer choices.^12^ These replacement words/phrases comprise the primary data for these analyses.

We also collected data on sociodemographic characteristics, including race/ethnicity, gender identity, sex assigned at birth, and sexual orientation. We measured gender identity with two questions: (1) an open-text field wherein participants could describe their gender identity in their own words and (2) a multiple-choice question asking participants to select all that apply from 12 gender-identity options.^14^ We asked sex assigned at birth with a multiple-choice question with four response options: “female,” “male,” “not listed (write-in),” and “prefer not to say.”^14^ Separately, we asked participants if they had ever received an intersex or difference in sex development diagnosis as well as whether they personally identified as intersex.

### Analysis

We conducted descriptive quantitative analyses to summarize data on respondent sociodemographic characteristics with Stata 15.1 (StataCorp, College Station, TX). We classified respondents into one of two gender categories based on analyses of responses to the gender identity and sex assigned at birth questions: (1) TGE and (2) CSMW.^14,15^ We excluded data from those whose gender identity was not classifiable (*n*=4).

Data cleaning involved eliminating explanations of term usage, separating multiple responses from a single participant so each registered as an individual response, and conversely, connecting multi-word responses with hyphens (e.g., “I use both shark week and bleeding when talking with friends” would become “shark-week” and “bleeding”). We did not alter the spelling of replacement words, as some misspellings may have been intentional. We included mentions of the medical term to capture respondents who stated they used multiple words, including the medical term, depending on context. We then imported cleaned responses into the qualitative analysis software MAXQDA (2020).^16^

In the first phase of analysis, we quantified the frequency with which respondents provided replacement words, and the most common words/phrases per medical term utilizing the Word Frequencies tool in MAXQDA. In the second phase of analysis, we employed a phenomenological qualitative content analysis approach^17^ to explore patterns and themes across replacement words. Specifically, we utilized the Word Cloud generator in MAXQDA to generate visual representations of replacement words such that larger terms reflect a higher number of mentions. We compared the word clouds and word frequency charts to identify patterns in the data, particularly around the use of “masculinized” words for those terms often associated with bodies assigned female at birth (e.g., “mancave” for “vagina”) and for words that expressed aversion to the medical term (e.g., “unwanted-chest” for “breasts”). To evaluate patterns in word-use by broad gender category (TGE and CSMW), we conducted several tests of proportion in Stata.

### Ethical review

This study was reviewed and approved by the Institutional Review Boards of Stanford University School of Medicine and the University of California, San Francisco. The PRIDE Study Research Advisory Committee and The PRIDE Study Participant Advisory Committee (PAC) (pridestudy.org) also reviewed and approved this study. All participants consented to participation before questionnaire initiation.

## Results

### Respondent characteristics

Overall, 5005 people initiated the questionnaire. Of these, 3074 people were classified as TGE (*n*=1704) or CSMW (*n*=1370) and responded to at least one of the nine two-part word-use questions. Respondents had a median age of 28 years (interquartile range: 23–35) and resided across all four U.S. Census regions (Table 1). Most respondents were white (87%) and college graduates (67%), and had health insurance (90%). A majority of respondents selected more than one gender identity (52%) and sexual orientation (59%). Most (99.7%) respondents reported their sex assigned at birth as female, and 0.3% (*n*=10) reported their assigned sex at birth as “not listed.” In a separate question, 3.2% (*n*=99) reported identifying as intersex.

**Table 1.** Sociodemographic Characteristics Among an Online Sample of Transgender, Nonbinary and Gender-Expansive (TGE) People and Cisgender Sexual Minority Women (CSMW) Recruited in the United States between May and September 2019

	TGE	TGE	CSMW	CSMW	Overall	Overall
	*n*=1704		*n*=1370		*n*=3074	
	27	(23–33)	30	(24–38)	28	(23–35)
Age median, (IQR)	*n*	%	*n*	%	*n*	%
Age (years)						
18–19	152	8.9	113	8.2	265	8.6
20–24	471	27.6	264	19.3	735	23.9
25–29	448	26.3	321	23.4	769	25.0
30–34	286	16.8	242	17.7	528	17.2
35–39	151	8.9	156	11.4	307	10.0
40–44	89	5.2	82	6	171	5.6
45–49	38	2.2	54	3.9	92	3.0
50–54	31	1.8	40	2.9	71	2.3
55–59	20	1.2	33	2.4	53	1.7
60–78	18	1.1	63	4.6	81	2.6
Missing	0	0	2	0.1	2	0.1
Gender identity						
Agender	227	13.3	1	0.1	228	7.4
Cisgender man	1	0.1	0	0	1	0.0
Cisgender woman	94	5.5	1150	83.9	1244	40.5
Genderqueer	658	38.6	0	0	658	21.4
Man	293	17.2	0	0	293	9.5
Nonbinary	875	51.3	0	0	875	28.5
Transgender man	664	39	0	0	664	21.6
Transgender woman	4	0.2	0	0	4	0.1
Two spirit	26	1.5	0	0	26	0.8
Woman	204	12	761	55.5	965	31.4
Additional gender	197	11.6	7	0.5	204	6.6
Missing	0	0	0	0	0	0.0
Prefer not to say GID	2	0.1	0	0	2	0.1
Selected multiple gender IDs	1039	61	543	39.6	1582	51.5
Sex assigned at birth						
Female	1694	99.4	1370	100	3064	99.7
Not listed	10	0.6	0	0	10	0.3
Intersex identification						
No	1613	94.7	1340	97.8	2953	96.1
Yes	70	4.1	29	2.1	99	3.2
Prefer not to say	21	1.2	1	0.1	22	0.7
Race/ethnicity						
American Indian/Alaskan Native	42	2.5	15	1.1	57	1.9
Black/African American	67	3.9	40	2.9	107	3.5
Central Asian	0	0	2	0.1	2	0.1
East Asian	41	2.4	33	2.4	74	2.4
Hispanic/Latinx	101	5.9	65	4.7	166	5.4
Middle Eastern, North African	24	1.4	16	1.2	40	1.3
Native Hawaiian, Pacific Islander	5	0.3	6	0.4	11	0.4
South Asian	19	1.1	11	0.8	30	1.0
Southeast Asian	25	1.5	15	1.1	40	1.3
White	1472	86.4	1201	87.7	2673	87.0
Missing race/ethnicity	89	5.2	68	5.0	157	5.1
None of these	4	0.2	2	0.1	6	0.2
Other	41	2.4	26	1.9	67	2.2
Selected multiple racial/ethnic identities	202	11.9	122	8.9	324	10.5
Unknown	12	0.7	3	0.2	15	0.5
Sexual orientation						
Asexual	252	14.8	111	8.1	363	11.8
Bisexual	571	33.5	583	42.6	1154	37.5
Gay	348	20.4	227	16.6	575	18.7
Lesbian	218	12.8	640	46.7	858	27.9
Pansexual	418	24.5	253	18.5	671	21.8
Queer	1150	67.5	641	46.8	1791	58.3
Questioning	69	4	37	2.7	106	3.4
Same-gender loving	111	6.5	99	7.2	210	6.8
Straight/hetero	61	3.6	5	0.4	66	2.1
Missing sexual orientation	31	1.8	10	0.7	41	1.3
Other sexual orientation	129	7.6	51	3.7	180	5.9
Selected multiple sexual orientations	1010	59.3	794	58	1804	58.7
Education level						
Some High School or less	23	1.3	14	1	37	1.2
High School degree or GED	118	6.9	66	4.8	184	6.0
Trade or tech school, but no degree	9	0.5	5	0.4	14	0.5
Trade or tech school degree	22	1.3	11	0.8	33	1.1
Some college, but no degree	379	22.2	195	14.2	574	18.7
College degree	519	30.5	376	27.4	895	29.1
Grad or professional study but no degree	125	7.3	106	7.7	231	7.5
Grad or professional degree	410	24.1	517	37.7	927	30.2
Missing	99	5.8	80	5.8	179	5.8
Health insurance coverage						
None	92	5.4	53	3.9	145	4.7
Yes	1512	88.7	1242	90.7	2754	89.6
Do not know	10	0.6	8	0.6	18	0.6
Missing	90	5.3	67	4.9	157	5.1
Census region						
Northeast	411	24.1	263	19.2	674	21.9
South	326	19.1	307	22.4	633	20.6
Midwest	304	17.8	263	19.2	567	18.4
West	468	27.5	368	26.9	836	27.2
Missing	195	11.4	169	12.3	364	11.8

CSMW, cisgender sexual minority women; IQR, interquartile range; TGE, transgender, nonbinary, and gender expansive.

### Use of replacement words overall

We present the most common replacement words by gender category and the distribution of medical term and replacement word usage in Table 2. Only two of the medical terms—“breasts” and “sperm”—had a single replacement word provided by more than 50% of those who provided replacement words: “chest” and “cum,” respectively. Otherwise, many (*n*=293, 23%) of the replacement words and phrases provided were unique.

**Table 2.** Use of Medical Terms and Top 3 Replacement Terms Among Transgender, Nonbinary, and Gender-Expansive (TGE) and Cisgender Sexual Minority Women (CSMW) Respondents in an Online Sample Recruited Between May and September 2019 in the United States, *n*=3074

		Word use	*n* (%)	Words	*n* (%)
Uterus	TGE respondents	Uses word	1664 (97.6)	Duderus	4 (14.81)
		Prefer not to say	12 (0.7)	Internal-junk	1 (3.7)
		Does not use word	28 (1.6)	All-that-(with-a-gesture)	1 (3.7)
	CSMW respondents	Uses word	1367 (99.8)	Womb	1 (50)
		Prefer not to say	1 (0.1)	Vagina	1 (50)
		Does not use word	2 (0.1)		
Vagina	TGE respondents	Uses word	1420 (83.5)	Front-hole	96 (33.1)
		Prefer not to say	31 (1.8)	Cunt	19 (6.6)
		Does not use word	250 (14.7)	Genitals	16 (5.5)
	CSMW respondents	Uses word	1359 (99.3)	Pussy	2 (16.7)
		Prefer not to say	1 (0.1)	Vagina	2 (16.7)
		Does not use word	9 (0.7)	Vulva	2 (16.7)
Period	TGE respondents	Uses word	1493 (87.8)	Bleeding	35 (15.6)
		Prefer not to say	15 (0.9)	Shark-week	33 (14.7)
		Does not use word	193 (11.3)	Cycle	26 (11.6)
	CSMW respondents	Uses word	1349 (98.5)	Menstruation	6 (27.3)
		Prefer not to say	2 (0.1)	Cycle	3 (13.6)
		Does not use word	18 (1.3)	Period	3 (13.6)
Breasts	TGE respondents	Uses word	1217 (71.5)	Chest	369 (72.4)
		Prefer not to say	18 (0.1)	Boobs	45 (8.8)
		Does not use word	466 (27.4)	Tits	14 (2.8)
	CSMW respondents	Uses word	1320 (96.4)	Boobs	35 (55.6)
		Prefer not to say	2 (0.1)	Tits	8 (12.7)
		Does not use word	47 (3.4)	Chest	6 (9.5)
Penis	TGE respondents	Uses word	1604 (94.5)	Dick	47 (45.2)
		Prefer not to say	13 (0.8)	Cock	24 (23.1)
		Does not use word	80 (4.7)	Penis	8 (7.7)
	CSMW respondents	Uses word	1349 (98.5)	Dick	11 (64.7)
		Prefer not to say	5 (0.4)	Cock	1 (5.9)
		Does not use word	15 (1.1)	Near-the-grain?	1 (5.9)
Sperm	TGE respondents	Uses word	1648 (97.1)	Cum	21 (55.3)
		Prefer not to say	17 (1)	Jizz	4 (10.5)
		Does not use word	32 (1.9)	Load	2 (5.3)
	CSMW respondents	Uses word	1349 (98.5)	Cum	2 (33.3)
		Prefer not to say	4 (0.3)	Jizzy-gene-swimmers	1 (16.7)
		Does not use word	6 (0.4)	Nasty	1 (16.7)
Birth control	TGE respondents	Uses word	1626 (96)	Contraception	24 (28.3)
		Prefer not to say	4 (0.2)	Contraceptive	6 (7.1)
		Does not use word	64 (3.8)	Contraceptives	6 (7.1)
	CSMW respondents	Uses word	1350 (98.6)	Contraception	7 (28)
		Prefer not to say	2 (0.1)	Birth-control	2 (8)
		Does not use word	17 (1.2)	Contraceptive	2 (8)
Pregnant	TGE respondents	Uses word	1677 (98.9)	Carrying-a-baby	2 (14.3)
		Prefer not to say	7 (0.4)	Knocked-up	2 (14.3)
		Does not use word	13 (0.8)	Preggers	2 (14.3)
	CSMW respondents	Uses word	1367 (99.9)	Maybe-too-complex	1 (100)
		Prefer not to say	1 (0.1)		
		Does not use word	1 (0.1)		
Abortion	TGE respondents	Uses word	1678 (99.1)	Termination	5 (35.7)
		Prefer not to say	4 (0.2)	Abortion	2 (14.3)
		Does not use word	12 (0.7)	Fetus-deletus	2 (14.3)
	CSMW respondents	Uses word	1356 (99.1)	Abortion	4 (28.6)
		Prefer not to say	4 (0.2)	Termination	4 (28.6)
		Does not use word	9 (0.6)	*D*&C	1 (7.1)

### Replacement words by gender category

Most respondents reported using the given medical terms. Specifically, 1057 (62%) TGE respondents and 1269 (93%) CSMW respondents reported using all nine medical terms. TGE respondents provided a higher number of replacement words (*n*=1138) as well as a higher proportion of respondents using at least one replacement word (*n*=613, 36%) compared to CSMW respondents (*n*=92, 7%; *p*-for-difference <0.001).

Among the 1704 TGE respondents, the most replaced words were “breasts” (*n*=466, 27%), followed by “vagina” (*n*=250, 15%) and “period” (*n*=193, 11%). These words also had the greatest diversity of responses with 62 unique responses provided for “breasts,” 92 for “vagina,” and 74 for “period.” Least replaced terms were “abortion” (*n*=12, 0.1%), “pregnant” (*n*=13, 0.1%), and “uterus” (*n*=28, 2%).

Among the 1370 CSMW respondents, replacement words were most commonly used for “breasts” (*n*=47, 3%), “period” (*n*=18, 1%), and “birth control” (*n*=17, 1%). These respondents least commonly replaced “pregnant” (*n*=1, 0.1%), “uterus” (*n*=2, 0.1%), and “sperm” (*n*=6, 0.4%).

**FIG. 1.**
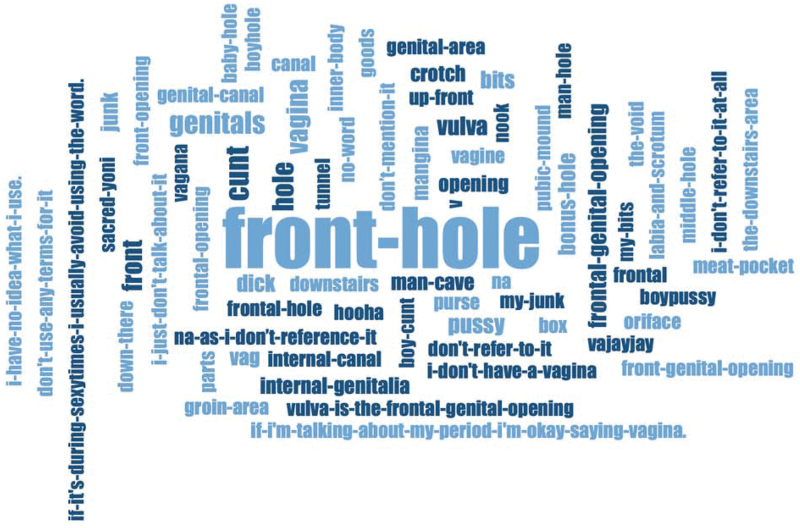
Word Cloud for replacement terms provided by TGE people for “vagina” within a national online sexual and reproductive health survey (May to September 2019, United States), *n*=1704. This figure shows the distribution of replacement words and phrases provided by TGE people for the word “vagina.” The size of the word or phrase reflects the number of TGE respondents who mentioned that they use this word or phrase. TGE, transgender, nonbinary, and gender expansive.

Although there is overlap in the words most and least replaced by CSMW and TGE respondents, the greatest difference in replacement word use occurred for “breasts” and “vagina,” anatomical words often associated with bodies assigned female at birth. TGE respondents were over 23 times more likely to replace “vagina” than CSMW respondents (Table 2; *p*-for-difference <0.001). An example word cloud for TGE replacement terms for “vagina” can be found in Figure 1. TGE respondents most commonly replaced “vagina” with the gender-neutral “front-hole,” comprising over 33% of the replacement words/phrases. Many TGE respondents also put some variation of “front-hole” (e.g., “frontal-hole”) (*n*=22, 8%) and “genitals,” (*n*=16, 6%). Six TGE respondents provided the word “dick,” and 13 provided a masculinized term (e.g., “mangina,” “mancave,” and “boypussy”); no CSMW respondents provided a neutral or masculinized replacement. Similarly, TGE respondents replaced the word “breasts” more than eight times as often as CSMW respondents (*p*-for-difference <0.001).

### Contextual replacement of and aversion to medical terms

Respondents in both gender categories provided contextual responses for replacement words, delineating when or with whom they might use a given word (Table 3). Specifically, five TGE respondents noted that the term “vagina” was either “too clinical” or that they would only use “vagina” in a medical context. Two participants reported using “vagina” either in a medical setting or to discuss their period, but avoiding that word during sex. For “breasts,” six respondents indicated that word-use depends on whose body is being discussed. For “period,” another wrote, “I use both; ‘period’ to doctors that don't understand transgender, or use ‘shark week’.”

**Table 3.** Contextual Responses Regarding If, When, and Why Participants Did or Did not Use A Medical Term, from Participants Recruited in an Online Sample in the United States Between May and September 2019, *n*=3074

	Contextual word-use (setting or person dependent)	Not applicable (does not talk about it, only talks about it to discuss removal, does not apply to respondent)	Does not have a word and/or discomfort with term
Uterus	“In reference to trans-masc friends, sometimes I use ‘duderus’”	“I just call it the stuff in there that I need to get removed” “I've never had a reason to think of what to call it honestly. The only time I ever bring up that area is when talking about a hysterectomy” “I don't have a uterus” “I don't have a uterus”	“When I must, I will say it, but use vague terminology. For example, ‘all that’ (with a gesture) to refer to reproductive system.”
Vagina	“For sexual activity I use the word hole. For medical appointments I use vagina” “In medical contexts I will use ‘vagina’ but socially if I am forced to refer to it I will just say ‘my junk’ or something’” “For medical reasons I use it but otherwise I pick any other word possible” “depends on the context-if it's during sexytimes I usually avoid using the word. If I'm talking about my period I'm okay saying vagina." "When referring to myself—‘front hole’ when referring to a woman-‘vagina’“ “Front hole (for myself)” “As long as not referring to my anatomy I use vagina”	“N/A” “I don't have a vagina”	“I have not referred to genitals since I've come/realized dysphoric/a. I have no idea what I use” “don't use any terms for it” “I just don't talk about it” “Don't mention it” “I have no idea what I use” “I haven't found a word that works for me that doesn't feel uncomfortable 'Down there’ or I don't refer to it at all N/A as I don't reference it I NEVER use this word, it causes me to dissociate to the point I can't walk. I don't use any terms for it.”
Period	“Medical vs. social distinction but doesn't come up often because I don't have one” “In a medical situation I will use the word “period” but socially I might say “shark week” or something although this doesn't come up often since I don't have one” “I use both period to doctors that don't understand transgender or use shark week” “I call my wife's period a period”	“I don't menstruate so I don't talk about it as something I have anymore” “I don't have a period”	“I do not talk about it or refer to it” “Never talk about it” “I do not use any word”
Breasts	“‘tits' if speaking casually, ‘breasts' if speaking formally. Also, ugh, flesh organs? What a horrifying description ☹ “ “I use “breasts,” “chest,” and “bust.” I also use “boobs” casually.” “breasts (technical), chest (polite company), boobs, boobies, titties, hugables... my girl and I have fun with them” “tits or boobs unless I'm in a medical setting” “when referring to other trans or cis guys-‘chest’” “Chest for myself” “chest (I had top surgery)” “As long as not referring to my pre-surgery anatomy breasts or boobs” “I use the word for others but I don't have them anymore” “I use ‘chest’ or ‘chesticles’. I don't like the word ‘breasts' used for my body”	“unwanted chest” “bullshit fleshbags removed from ur body” “I don't have breasts”	“boobs, breasts makes me anxious and uncomfortable for whatever reason”
Penis	“'Dick’ if talking casual, penis if speaking formally.” “Only for medical term—any slang term for other times” “Penis or dick or cock or whatever the person whose body it's attached to calls it.” “Sometimes penis, but if it's a cisman's organ, I might refer to it as his dick. If I'm talking about my body I likely say cock.” “Dickoris for AFAB clitoris after testosterone-induced growth” “My partner is trans feminine and we use the word clit” “I use penis when describing cismale genitals, but I do not use penis to describe my own genitals, instead I use ‘my genitals’”	“I have a clit and call it that” “Don't typically talk about penises” “I actually don't have the need to say penis. So I think when I do, I say ‘pee pee’. I am struggling to recall the last time I said penis.”	
Sperm		“don't usually talk about sperm”	

In response to six of the nine medical terms (“breasts,” “penis,” “period,” “sperm,” “uterus,” and “vagina”), 34 TGE respondents (2%) stated that the term did not apply to them, they did not have a word, or they avoid talking about the subject. For “vagina,” nine respondents reported they do not have a word or never reference a “vagina.” Notably, one respondent wrote “I NEVER use this word, it causes me to dissociate to the point I can't walk. I don't use any terms for it.” Five respondents reported not having breasts, having had top surgery, or associating breasts with their removal (e.g., “bullshit flesh bags removed from ur [sic] body”). Similarly, for “uterus,” one respondent explained, “the only time I ever bring up that area is when talking about a hysterectomy.”

CSMW respondents provided contextual responses and responses that expressed aversion with similar frequency to TGE respondents (*p*=0.66 and *p*=0.38). For “breasts,” one CSMW respondent stated they would use “tits or boobs, unless I'm in a medical setting”; another explained, “‘breasts' makes me anxious and uncomfortable for whatever reason.”

## Discussion

### Key findings

These results from a national survey of TGE people and CSMW assigned female or intersex at birth highlight several important findings. First, more than one in three respondents (36%) reported not using at least one medical term. Second, TGE respondents were more likely to replace words, with the greatest difference occurring for body parts often associated with bodies assigned female at birth: “vagina” and “breasts.” Third, the replacement words varied widely; almost a quarter were unique. Fourth, many respondents delineated word-use by context, indicating (1) that word-use is dependent on setting and (2) an assumption that health care providers may not understand words that reflect the experiences of SGM people. Finally, many respondents expressed discomfort with certain terms and explained that some terms did not apply to them.

### Limitations and strengths

As with all research, this study had limitations. As no comprehensive sampling frame of TGE people exists, nor one for CSMW, we relied on convenience sampling; thus, our sample may not be representative of these populations. Furthermore, while 22% of respondents identified with a non-white racial/ethnic identity, our low number of respondents who identify as Black/African American, Hispanic/Latinx, or another racial minority does not adequately reflect the racial diversity that exists among SGM people. In addition, we recognize the limitations of comparing TGE individuals with CSMW. This is not to imply that CSMW serve as an adequate comparison population nor representative of the general population, rather to reflect on potentially different experiences (e.g., transphobia) in clinical and research settings.

Specific to word-use data, while many participants explained why or when they use a specific word, we did not systematically measure word-use by context. Consequently, we cannot say whether these replacement words would or would not be acceptable for providers to use in clinical settings (as respondents may have provided them only as words that they feel comfortable using for their own bodies in private settings). Relatedly, in one study on provider communication preferences of transgender men and trans-masculine nonbinary individuals, most respondents reported preferring that providers use medical terms rather than slang when referring to their bodies.^11^ Future research should ask participants what words they use for a given range of contexts and why.

Separately, our gender-neutral definitions for each SRH medical term, intentionally created with feedback from community stakeholders, may have influenced the desire to provide replacement words. We cannot know if or how replacement word-use might vary with standard medical definitions or those derived in another context, compared to the study-specific, gender-neutral definitions we provided in this survey. Because we decided to offer all respondents the opportunity to provide their own words, we cannot assess whether this feature influenced survey retention.

Specific to data cleaning, because we did not alter spelling to capture potential alternate spellings of words, we may have missed patterns across words that were unintentionally misspelled. As MAXQDA tools were not case sensitive, differences in use of caps and hyphens were not captured.

These limitations are balanced by strengths, namely the innovative survey design that allowed respondents to customize the language of the survey, and the large sample size spanning multiple gender identity categories, ages, and regions.

## Conclusions

The diversity of replacement words provided by respondents emphasizes the importance of avoiding assumptions about the SRH words that people use.^11^ The many contextual responses suggest that some individuals may not always use their preferred word for safety, to avoid having to explain themselves, or because of assumed nonacceptability in certain (primarily clinical) contexts. These findings add context to prior research findings that the communication skills and language used by health care providers can affect if and to what extent patients disclose relevant details about their sexual orientations and gender identities.^3,11,18,19^

These findings emphasize the importance of person-centered language, mirroring an individual's own language, specifically inviting each individual to provide the word(s) they use to refer to a particular body part or process. Using person-centered language addresses issues of discrimination as individuals may associate inaccurate language, including the use of incorrect pronouns, with negative attitudes toward them on the part of providers.^20–22^ In addition, defining words and then inviting individuals to provide their own may allow for the provision of more accurate and responsive clinical care and research, as we saw many respondents provided replacement words that applied to different body parts, expressed strong aversion to certain terms, or expressed that a given term did not apply to them.

To guide interactions between health care providers and patients, and between researchers and participants, it is important to clearly state the rationale for asking certain questions (and to ensure each question is necessary before asking it).^23^ For example, “In order to assess your risk of X, I am going to ask you a few questions about Y.” To get on the same page about the words being used, a provider or researcher could say “This is the word I use to talk about Z. What word do you use?” In the case of written research materials, investigators could adopt a similar piped-in language approach to the customizable survey used in this study. The full text of this survey and associated methodology has been published elsewhere.^12^

Cisnormative and heteronormative language have been a source of great discrimination, erasure, and oppression for SGM people, contributing to health disparities in SRH. We offer these findings in the hope that others will adopt and adapt these methods to create more inclusive and individualized clinical and research experiences, which place greater autonomy in the hands of patients and research participants. These findings offer insight into the diversity of ways people talk about their bodies, and the impact on patients and research participants of imposing language that may carry cisnormative assumptions. Our work highlights the importance of avoiding assumptions, creating as much opportunity as possible to use person-centered language to eliminate potential gaps in knowledge, care, and measurement, and, most importantly, to ensure that we use affirming language that promotes health rather than harm.

## Abbreviations Used

CSMW: cisgender sexual minority women
IQR: interquartile range
NSFG: National Survey of Family Growth
PAC: Participant Advisory Committee
PRIDE: Population Research in Identity and Disparities for Equality
SGM: sexual and gender minority
SRH: sexual and reproductive health
TGE: transgender, nonbinary, and gender expansive

## References

[1] Thomas L, Wareing S. Language, Society and Power. London, UK: Routledge, 2004.

[2] Zimman L. Transgender language reform. J Lang Discrimination. 2017;1:84–105.

[3] Hagen DB, Galupo MP. Trans* individuals' experiences of gendered language with health care providers: recommendations for practitioners. Int J Transgender. 2014;15:16–34.

[4] Baldwin A, Dodge B, Schick VR, et al. Transgender and genderqueer individuals' experiences with health care providers: what's working, what's not, and where do we go from here? J Health Care Poor Underserv. 2018;29:1300–1318. 10.1353/hpu.2018.0097 PMC10371218 30449748

[5] Center for Health Statistics N. 2015-2017 NSFG FEMALE CAPI-Lite 2015-2017 National Survey of Family Growth FEMALE Questionnaire. Hyattsville, Maryland: Center for Health Statistics, 2015

[6] Moseson H, Zazanis N, Goldberg E, et al. The imperative for transgender and gender nonbinary inclusion. Obstet Gynecol. 2020;135:1059–1068. 32282602 10.1097/AOG.0000000000003816 PMC7170432

[7] Carrotte ER, Vella AM, Bowring AL, et al. I am yet to encounter any survey that actually reflects my life: a qualitative study of inclusivity in sexual health research. BMC Med Res Methodol. 2016;16:86. 27465507 10.1186/s12874-016-0193-4 PMC4964098

[8] Wingo E, Ingraham N, Roberts SCM. Reproductive health care priorities and barriers to effective care for LGBTQ people assigned female at birth: a qualitative study. Womens Health Issues. 2018;28:350–357. 29661698 10.1016/j.whi.2018.03.002

[9] Stroumsa D, Wu JP. Welcoming transgender and nonbinary patients: expanding the language of “women's health.” Am J Obstet Gynecol. 2018;219:585.e1–e585.e5. 30267652 10.1016/j.ajog.2018.09.018

[10] Hoffkling A, Obedin-Maliver J, Sevelius J. From erasure to opportunity: a qualitative study of the experiences of transgender men around pregnancy and recommendations for providers. BMC Pregnancy Childbirth. 2017;17(S2):332. 29143629 10.1186/s12884-017-1491-5 PMC5688401

[11] Klein A, Golub SA. Enhancing gender-affirming provider communication to increase health care access and utilization among transgender men and trans-masculine non-binary individuals. LGBT Health. 2020;7:292–304. 32493100 10.1089/lgbt.2019.0294 PMC7475086

[12] Moseson H, Lunn MR, Katz A, et al. Development of an affirming and customizable electronic survey of sexual and reproductive health experiences for transgender and gender nonbinary people. Micks EA, ed. PLoS One. 2020;15:e0232154. 32365110 10.1371/journal.pone.0232154 PMC7197812

[13] Lunn MR, Lubensky M, Hunt C, et al. A digital health research platform for community engagement, recruitment, and retention of sexual and gender minority adults in a national longitudinal cohort study—the PRIDE study. J Am Med Inform Assoc. 2019;26:737–748. 31162545 10.1093/jamia/ocz082 PMC6696499

[14] Tate CC, Ledbetter JN, Youssef CP. A two-question method for assessing gender categories in the social and medical sciences. J Sex Res. 2013;50:767–776. 22989000 10.1080/00224499.2012.690110

[15] The GenIUSS Group. Best Practices for Asking Questions to Identify Transgender and Other Gender Minority Respondents on Population-Based Surveys. Available at https://escholarship.org/uc/item/3qk7s1g6 Accessed August 2, 2020.

[16] MAXQDA 2020 New Features | Jump Start Your Research Journey-MAXQDA. Available at https://www.maxqda.com/new-maxqda-2020 Accessed August 24, 2020.

[17] Kuckartz U. Qualitative Text Analysis: A Systematic Approach. Cham: Springer, 2019, pp. 181–197.

[18] Glick JL, Theall K, Andrinopoulos K, et al. For data's sake: dilemmas in the measurement of gender minorities. Cult Health Sex. 2018;20:1362–1377. 29533145 10.1080/13691058.2018.1437220 PMC6526522

[19] Brooks H, Llewellyn CD, Nadarzynski T, et al. Sexual orientation disclosure in health care: a systematic review. Br J Gen Pract. 2018;68(668):e187–e196. 29378698 10.3399/bjgp18X694841 PMC5819984

[20] Shelton K, Delgado-Romero EA. Sexual orientation microaggressions: the experience of lesbian, gay, bisexual, and queer clients in psychotherapy. J Counsel Psychol. 2011;58:210–221. 10.1037/a0022251 21463031

[21] Brown C, Frohard-Dourlent H, Wood BA, et al. “It makes such a difference.” J Am Assoc Nurse Pract. 2020;32:70–80. 31232865 10.1097/JXX.0000000000000217

[22] Fuzzell L, Fedesco HN, Alexander SC, et al. “I just think that doctors need to ask more questions”: sexual minority and majority adolescents' experiences talking about sexuality with healthcare providers. Patient Educ Counsel. 2016;99:1467–1472. 10.1016/j.pec.2016.06.004 27345252

[23] Suen LW, Lunn MR, Katuzny K, et al. What sexual and gender minority people want researchers to know about sexual orientation and gender identity questions: a qualitative study. Arch Sex Behav. 2020;49:2301–2318. 32875381 10.1007/s10508-020-01810-y PMC7497435

